# Kasabach-Merritt Syndrome in an Adult

**DOI:** 10.4274/tjh.galenos.2019.2019.0068

**Published:** 2020-02-20

**Authors:** Milan Pantelic, Masa Pantelic, Petar Djuric, Katarina Markovic, Tamara Vucinic, Jovan Todor Juloski

**Affiliations:** 1 Zvezdara University Medical Center, Department of Radiology, Belgrade, Serbia; 2 Zvezdara University Medical Center, Department of Gastroenterology, Belgrade, Serbia; 3 Zvezdara University Medical Center, Department of Nephrology, Belgrade, Serbia; 4 Zvezdara University Medical Center, Department of Surgery, Belgrade, Serbia; 5 Zvezdara University Medical Center, Department of Surgery, Belgrade, Serbia

**Keywords:** Kasabach-Meritt syndrome, Vascular lesion, Retroperitoneal tumor, Multislice computed tomography

Kasabach-Merritt syndrome (KMS) is a vascular disease characterized by the presence of thrombocytopenia, anemia, disseminated intravascular coagulation (DIC), and vascular lesions. It was first described in 1940 by Kasabach-Merritt [1,2,3]. KMS often occurs during infancy and the neonatal period and rarely in adults [1,2,4]. KMS is commonly associated with kaposiform hemangioendothelioma and tufted angioma, which are rare vascular tumors produced by the lymph and capillary endothelium with positive immunohistochemical staining for vascular markers (CD31, CD34) and focal positivity for lymphatic markers (LYVE1, PROKS1, D2-40), while they are negative for GLUT1 and Lewis Y antigen (markers specific to hemangiomas) [1,5].

A 22-year-old woman presented to the emergency department with abdominal pain, fever, and vomiting. Laboratory evaluation showed moderate anemia, DIC (hypofibrinogenemia, thrombocytopenia, prolonged prothrombin, and activated partial thromboplastin time), and elevated D-dimer. Multislice computed tomography showed a large retroperitoneal tumor (Figures 1, 2). An exploratory laparotomy with biopsy was performed (Figure 3). Histopathological examination revealed a vascular lesion (positive for CD31 and CD34 positive; focally positive for D2-40). The patient was transferred to another hospital where she was treated with corticosteroids (prednisone at 40 mg daily), fresh frozen plasma, and cryoprecipitate, which led to an improvement. After six months, magnetic resonance imaging showed a regression in tumor size.

## Figures and Tables

**Figure 1:**
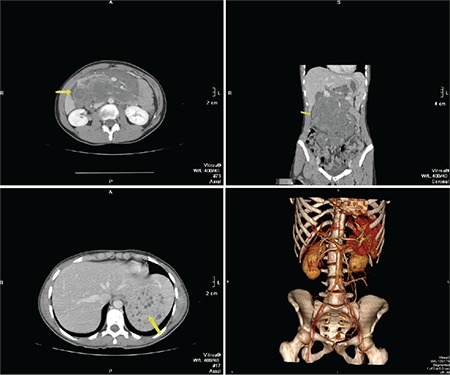
Multislice computed tomography showed a large retroperitoneal tumor.

**Figure 2:**
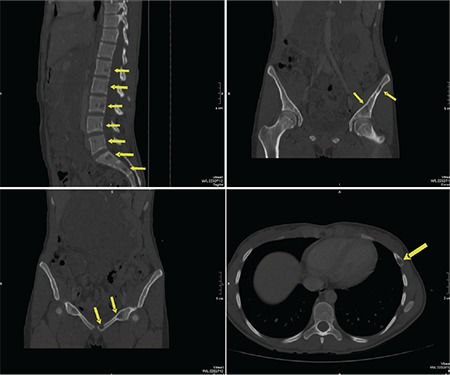
Multislice computed tomography showed a large retroperitoneal tumor.

**Figure 3:**
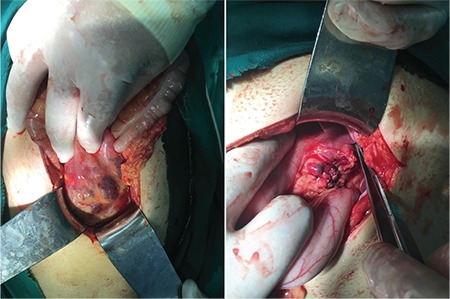
An exploratory laparotomy with biopsy was performed.
